# Impact of the griffithsin anti-HIV microbicide and placebo gels on the rectal mucosal proteome and microbiome in non-human primates

**DOI:** 10.1038/s41598-018-26313-8

**Published:** 2018-05-23

**Authors:** Lauren Girard, Kenzie Birse, Johanna B. Holm, Pawel Gajer, Mike S. Humphrys, David Garber, Patricia Guenthner, Laura Noël-Romas, Max Abou, Stuart McCorrister, Garrett Westmacott, Lin Wang, Lisa C. Rohan, Nobuyuki Matoba, Janet McNicholl, Kenneth E. Palmer, Jacques Ravel, Adam D. Burgener

**Affiliations:** 1 National HIV and Retrovirology Labs, JC Wilt Infectious Diseases Research Centre, Public Health Agency of Canada, Winnipeg, Canada; 2 Department of Medical Microbiology, University of Manitoba, Winnipeg, Canada; 3 Institute for Genome Sciences, University of Maryland School of Medicine, Baltimore, USA; 4 Department of Microbiology and Immunology, University of Maryland School of Medicine, Baltimore, USA; 5 Laboratory Branch, Division of HIV/AIDS Prevention, National Centre for HIV/AIDS, Viral Hepatitis, Sexually Transmitted Disease and Tuberculosis Prevention, CDC, Atlanta, USA; 6 Mass Spectrometry and Proteomics Core Facility, National Microbiology Laboratory, Public Health Agency of Canada, Winnipeg, Canada; 7 Magee Women’s Research Institute, Pittsburgh, USA; 8 School of Pharmacy, University of Pittsburgh, Pittsburgh, USA; 9 Center for Predictive Medicine, University of Louisville, Louisville, USA; 10 Department of Pharmacology and Toxicology, University of Louisville, Louisville, USA; 11 James Graham Brown Cancer Centre, University of Louisville, Louisville, USA; 12 Unit of Infectious Diseases, Department of Medicine Solna, Centre for Molecular Medicine, Karolinska Institute, Karolinska University Hospital, Stockholm, Sweden

## Abstract

Topical microbicides are being explored as an HIV prevention method for individuals who practice receptive anal intercourse. *In vivo* studies of these microbicides are critical to confirm safety. Here, we evaluated the impact of a rectal microbicide containing the antiviral lectin, Griffithsin (GRFT), on the rectal mucosal proteome and microbiome. Using a randomized, crossover placebo-controlled design, six rhesus macaques received applications of hydroxyethylcellulose (HEC)- or carbopol-formulated 0.1% GRFT gels. Rectal mucosal samples were then evaluated by label-free tandem MS/MS and 16 S rRNA gene amplicon sequencing, for proteomics and microbiome analyses, respectively. Compared to placebo, GRFT gels were not associated with any significant changes to protein levels at any time point (FDR < 5%), but increased abundances of two common and beneficial microbial taxa after 24 hours were observed in HEC-GRFT gel (p < 2E-09). Compared to baseline, both placebo formulations were associated with alterations to proteins involved in proteolysis, activation of the immune response and inflammation after 2 hours (p < 0.0001), and increases in beneficial *Faecalibacterium* spp. after 24 hours in HEC placebo gel (p = 4.21E-15). This study supports the safety profile of 0.1% GRFT gel as an anti-HIV microbicide and demonstrates that current placebo formulations may associate with changes to rectal proteome and microbiota.

## Introduction

Receptive anal intercourse (RAI) is a large contributing factor to new HIV infections globally. Specifically, HIV transmission risk during condom-less receptive anal intercourse (CRAI) is estimated to be 138 per 10,000 exposures (95% CI 102-186 per 10,000)^1^. RAI is commonly practiced by men who have sex with men (MSM), who continue to be disproportionately affected by the global HIV epidemic^2,3^, accounting for 49% of new infections in Western and Central Europe and North America, 30% of new infections in Latin America, and 18% of new infections in Asia and the Pacific according to the most recent UNAIDS Global Report^4^. CRAI may also be contributing to HIV infection rates observed in other key populations, such as transgender women, in which HIV prevalence is 19.1% worldwide, as well as women in sub-Saharan Africa^5,6^. Despite this high risk of acquisition, the level of condom use among those practicing RAI is inadequate^7–16^; demonstrating the importance of providing novel HIV prevention tools for these populations is critical for stemming the rate of new infections.

Microbicides are topical antiviral compounds used to prevent HIV transmission at mucosal surfaces. Since people who practice RAI often use sexual lubricants, a microbicide incorporated into a gel-based lubricant may represent a practical and acceptable prevention method against rectal transmission of HIV^17^. An essential step in the development of new microbicide candidates is the determination of their effects on biological systems. Microbicides that elicit undesirable side effects may impact tolerance and adherence to the product. Notably, certain microbicides may elicit biological responses that lead to increased risk of HIV acquisition. The importance of pre-clinical toxicity screening has been demonstrated by the early microbicide candidate, Nonoxynol-9 (N-9). Although N-9 was presumed safe and used as a spermicide, it was shown to cause increased genital lesions, increased inflammation, toxicity and increased risk for HIV acquisition^18,19^. Recent studies have highlighted the significance of mucosal inflammation on increased HIV acquisition risk^20^. In addition, N-9 delivered intra-vaginally shifted the microbiota toward a state of lowered *Lactobacillus* spp. levels and increases in strict and facultative anaerobes, which is a known risk factor for HIV acquisition and transmission^21–23^. However, there is limited information of rectal inflammation or microbiome parameters that lead to increased HIV acquisition.

Currently, the lead rectal microbicide candidate is Tenofovir (TFV) 1% gel, with a phase II trial (MTN-017) currently underway^24^. Despite this progress, there are safety concerns related to the topical use of TFV. A study on the long-term use of TFV 1% gel demonstrated a broad range of effects on rectal mucosa, including suppressed anti-inflammatory mediators, increased T cell densities, mitochondrial dysfunction, altered regulatory pathways of cell differentiation and survival, and stimulation of cell proliferation^25^. Additionally, in a recent study by Romas *et al*., rectal TFV 1% gel use was associated with altered epidermal protein expression^26^. While the significance of these alterations with HIV acquisition risk and gel efficacy are currently unknown, efforts to develop safe rectal microbicide that do not impact the natural environment of the rectal mucosa are desirable.

Another important consideration in the development of new microbicide products is the choice of gel formulation^27^. Gel formulation determines the characteristics of the gel product, which include gel appearance, pH, osmolality and viscosity. In turn, these features can influence the safety of and the tolerability to the product. The importance of gel formulation was demonstrated by the use of glycerin as an excipient in the RMP-02/MTN-006 and the MTN-007 clinical trials. Notably, glycerin is commonly used in gel products in order to increase solubility of active ingredients. Conversely, it has been suggested that the hyper-osmolar properties of glycerin may induce changes in the rectal compartment, leading to enhanced HIV-1 infection^28,29^. In contrast to RMP-02/MTN-006, MTN-007 used a gel product containing a reduced amount of glycerin. This change in formulation was associated with fewer adverse events^30,31^, demonstrating the importance of exploring multiple gel formulations in the development of rectal microbicide products.

PREVENT (PREvention of Viral ENTry) is a placebo-controlled crossover preclinical trial with the goal of evaluating the tolerability and safety of GRFT, a potent antiviral lectin, as a rectal microbicide to prevent HIV infection. Past studies have shown GRFT’s safety in various systems, including no cytotoxicity in several different cell lines, including cervical (End1/E6E7, Ect1/E6E7, CaSki), fibroblast (3T3) and dendritic (moDC) cell lines^32,33^. These studies also showed that GRFT was associated with little or no induction of inflammatory cytokines in both human cervical explant tissues and Peripheral Blood Mononuclear Cells (PBMCs), no induction of T-cell activation, no off-target alterations in gene expression^32–35^ and demonstrated safety in rodent models and rabbit vaginal irritation studies^35,36^.

The recent emergence of systems biology tools, such as proteomics and transcriptomics, has made available new tools to study mucosal systems. These techniques have provided insight into mechanisms of mucosal system perturbations during a wide variety of mucosal inflammatory conditions, including vaginal microbial dysbiosis^37^, elevated mucosal cytokines^38^, effects of hormonal contraceptives^39^, and the effects of sexual behaviours, such as CRAI^40^. Recently, systems biology techniques demonstrated the off-target effects of a tenofovir-based microbicide gel on rectal compartments, proving the utility of this method to screen potential toxic effects of anti-retroviral based microbicides^26^. In this study, we evaluated the rectal mucosal proteome, as well as the composition and structure of the rectal microbiota associated with intra-rectal application of 0.1% GRFT gel, while comparing two different gel formulations, HEC and Carbopol, in a non-human primate (NHP) model.

## Results

### Study design

**Figure 1:**
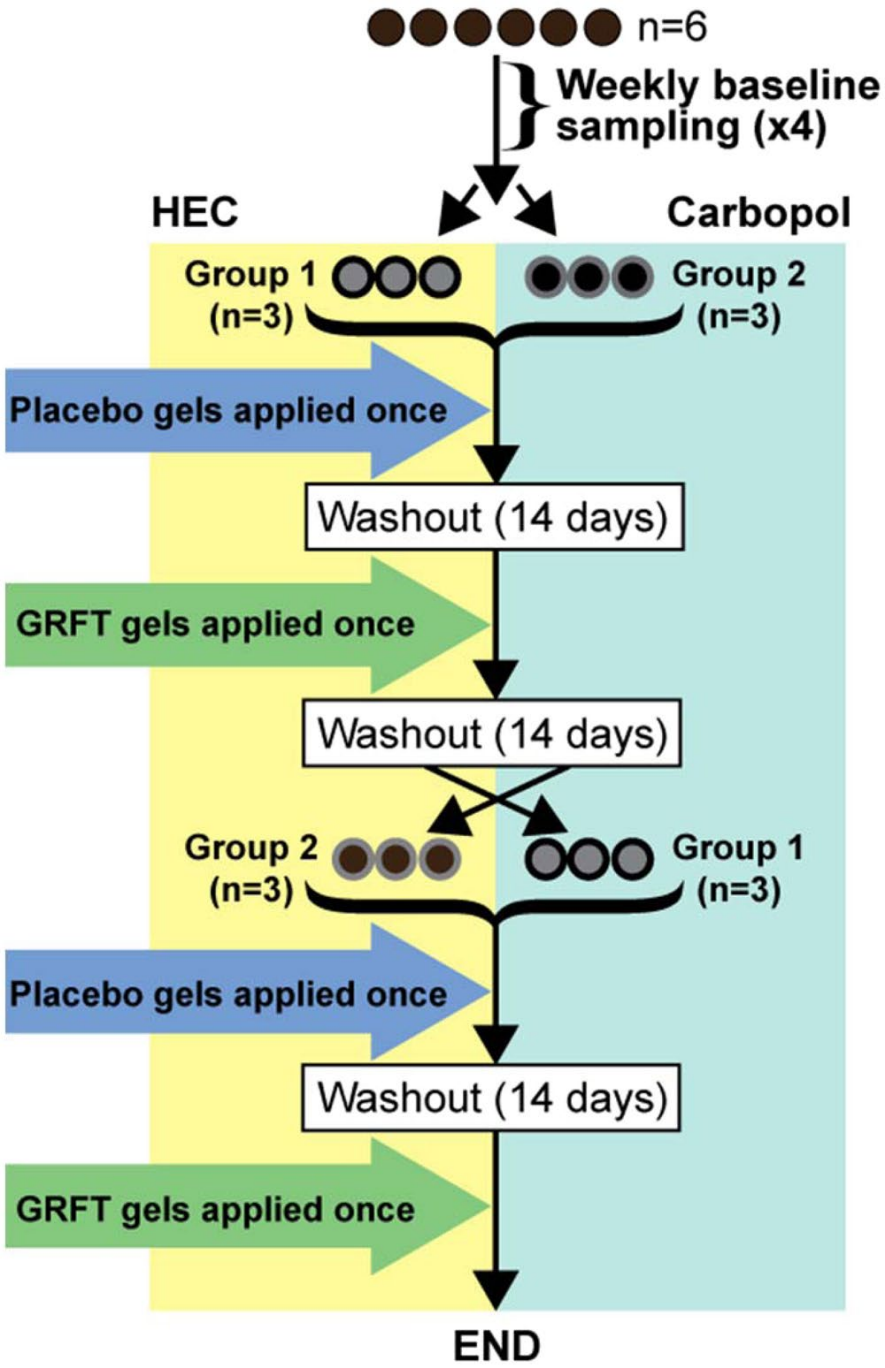
An outline of the PREVENT study in rhesus macaques. Baseline samples were collected from six rhesus macaques prior to dividing the macaques into two groups (Group 1 and Group 2). Macaques were then treated once intra-rectally with placebo gels, followed by a washout period of 14 days, and subsequently treated intra-rectally with GRFT 0.1% gels. The formulation applied was dependent on group assignment. After another 14-day washout period, there was a mid-trial crossover point in which the two groups switched gel formulations. The placebo and 0.1% GRFT gel application process was repeated.

Rhesus macaques were maintained at the Centers for Disease Control and Prevention (CDC; Atlanta, GA, USA) in accordance with the Guide for the Care and Use of Laboratory Animals (8^th^ edition) in an AALAC-accredited facility, according to institutional standard operating procedures. All study methods and procedures were approved by the CDC Institutional Animal Care and Use Committee (IACUC, protocol 2700GARMONC). Six rhesus macaques were split into two groups and treated intra-rectally with either HEC or carbopol formulations of both 0.1% GRFT and placebo gels, followed by a formulation crossover after a two-week washout period (Fig. 1). Multiple (two or four samples for for microbiota and proteomic studies, respectively) longitudinal rectal swabs were collected over a period of 4 weeks from all six macaques prior to gel initiation to establish robust baseline protein abundance measurements. Each gel application was preceded by collection of pre-gel (T0) rectal swabs and followed by collection of post-gel rectal swabs (2 hours and 24 hours post-gel application for proteomic analysis; 24 hours and 7 days for microbiota analysis). These samples were then analysed by tandem mass spectrometry and 16 S rRNA gene amplicon sequencing.

### Mass spectrometry analysis and proteome coverage of rectal mucosal samples from rhesus macaques

We first evaluated rectal sample proteome variability and reproducibility by mass spectrometry. Mass spectrometry analysis confidently identified 382 host proteins across all baseline, placebo-treated and GRFT-treated samples. Total protein abundance was visualized to identify sample outliers (Supplementary Fig. S1). Nine technical replicates of a reference sample (containing equal amounts of peptide from each rectal mucosal sample) were used to evaluate the reproducibility of the mass spectrometer. This comparison showed strong correlation of individual proteins between sample runs, with Pearson r values > 0.985 (Supplementary Fig. S2). This demonstrated high fidelity of mass spectrometry to profile rectal mucosal samples.

**Figure 2:**
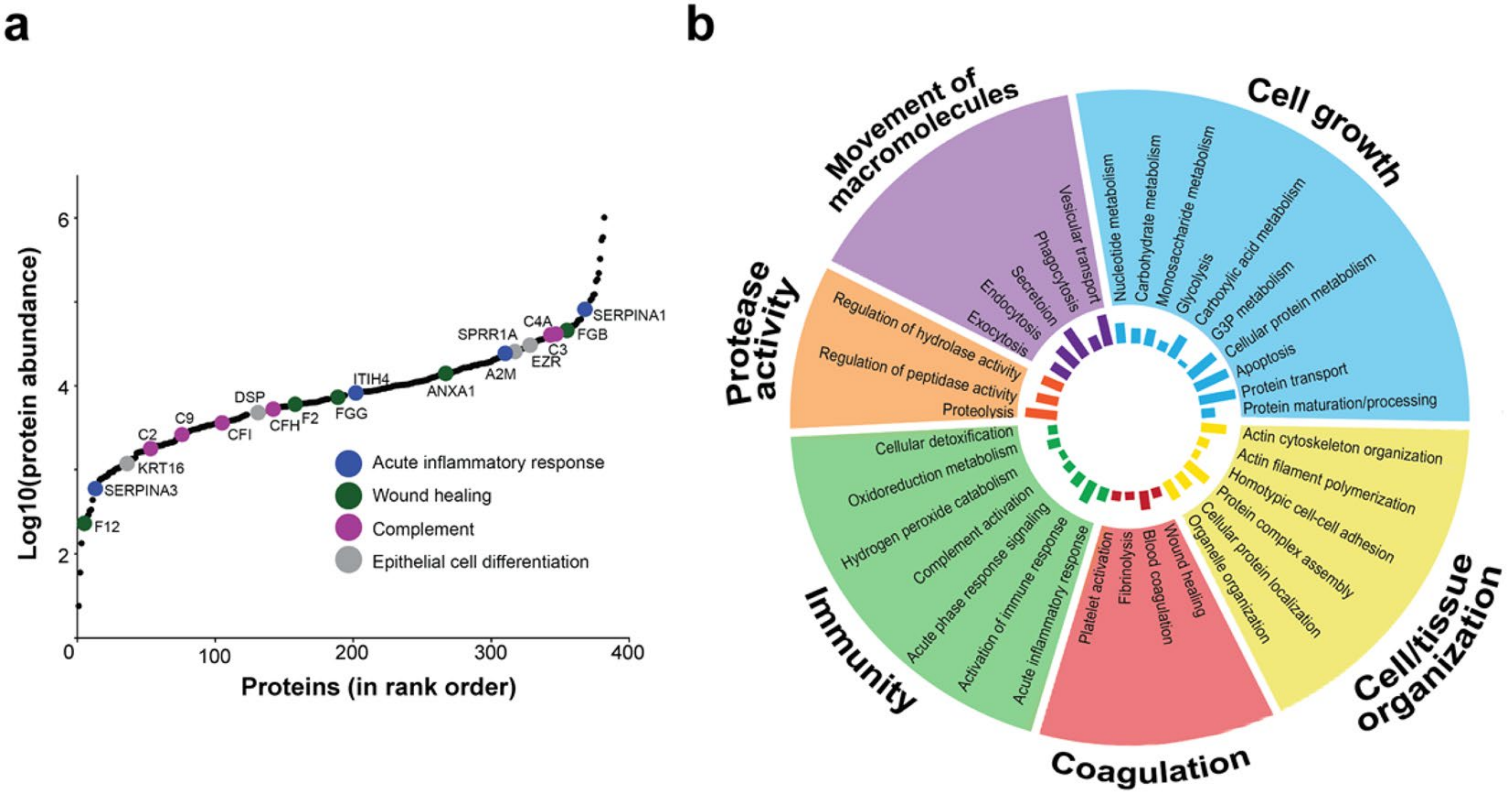
Diversity of the rectal mucosal proteome observed by mass spectrometry. (**a**) Log Rank plot of protein abundances shows rectal proteome coverage spans greater than 4 orders of magnitude. Immune proteins belonging to specific pathways are indicated in dark blue (acute inflammatory response), dark green (wound healing), pink (complement) and grey (epithelial cell differentiation); (**b**) Radial chart of the top biological processes in the rectal mucosa of rhesus macaques in our study according to DAVID Functional Annotation v6.8. The innermost bar chart represents the number of proteins involved in each process. Processes are grouped into broader categories, which include cell growth, cell/tissue organization, coagulation, immunity, protease activity and movement of macromolecules.

The abundance of proteins detected in rectal mucosa spanned over 4 orders of magnitude (Fig. 2a). According to gene ontology and pathway analysis, the top biological processes associated with these proteins included in cell growth, cell/tissue organization, coagulation, immunity, protease activity and movement of macromolecules (Fig. 2b). Functional annotation ascribed rectal proteins to several cellular compartments, including the cytoplasm (p = 3.12E-32), extracellular vesicles (p = 1.54E-187), intracellular organelles (p = 2.82E-066), adherens junctions (p = 7.81E-40), the cytoskeleton (p = 4.16E-10) and blood microparticles (p = 5.17E-40) (Supplementary Fig. S3). Overall this shows that a diverse set of biological functions and pathways are observable in rectal mucosal samples by mass spectrometry.

### Transient host proteome changes are associated with HEC and Carbopol placebo gel use

Four baseline samples were taken and protein abundances from these samples were averaged to establish a baseline proteome measurement for each animal (shown in Supplementary Fig. S4). Further visualization by Principle Component Analysis showed that baseline averages were less variable than samples collected at other time points (Supplementary Fig. S5).

**Figure 3:**
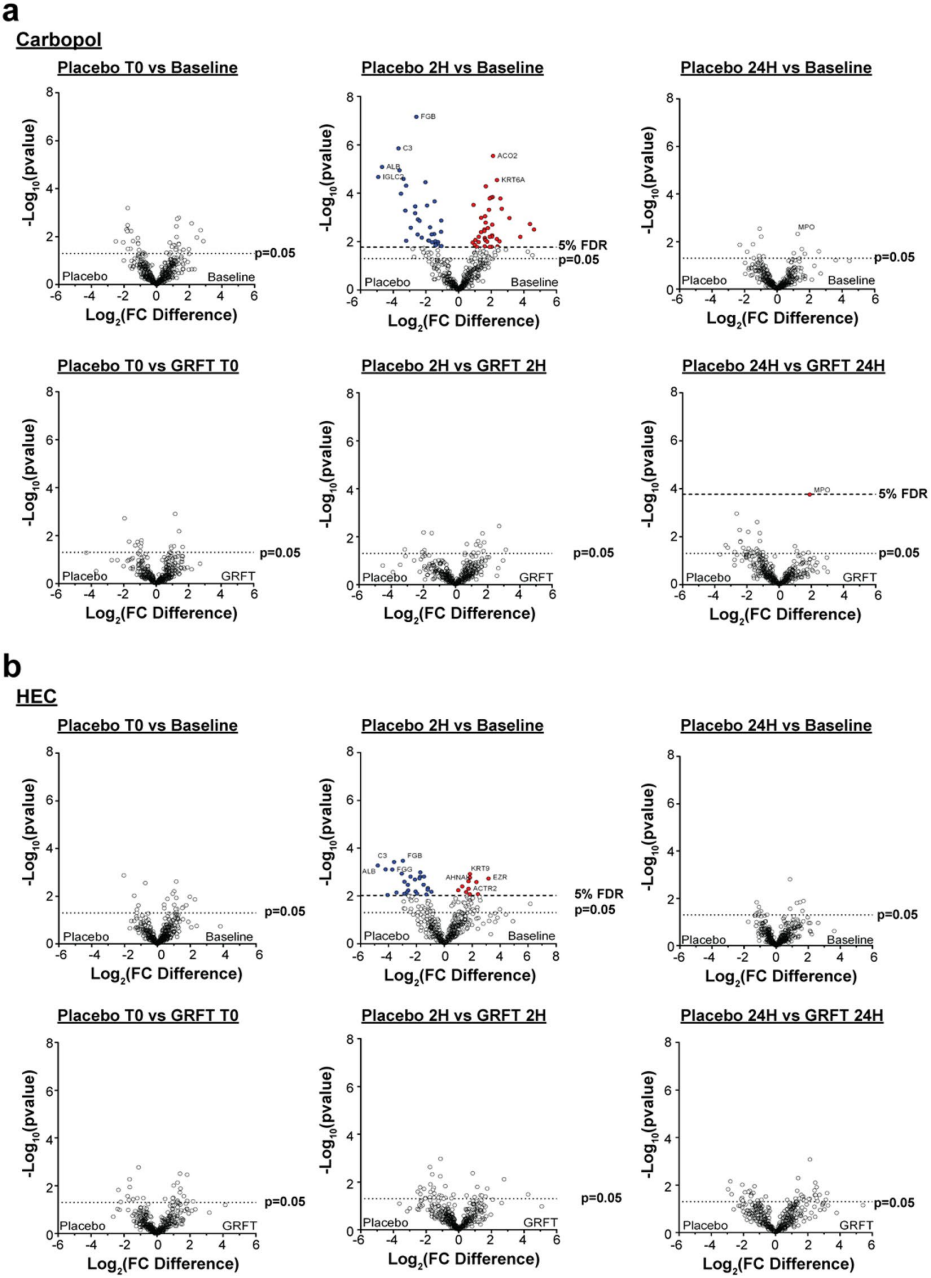
Changes to the rectal mucosal proteome after application of either drug (0.1% GRFT) or placebo using either hydroxyethylcellulose (HEC) or carbopol formulations. Volcano plots display the log2-fold change values of protein expression along the x-axis and −log p-value (as determined by paired *t* tests) along the y-axis. Dashed lines indicate the cut off for p = 0.05 and the 5% false discovery rate (FDR) where applicable. Blue and red points represent proteins that were significantly over- and under-abundantly expressed in placebo-treated samples, respectively (threshold < 5% FDR). Significant effects were observed 2 hours post-placebo gel application for both carbopol and HEC gels, where 19.9% and 9.4% of proteins were differentially expressed, respectively (upper middle panels of **a** and **b**). Other than relatively higher levels of Myeloperoxidase at 24 hours post-GRFT 0.1% gel application in the carbopol formulation (**a**, bottom right), 0.1% GRFT gels did not elicit any significant changes in rectal mucosal protein expression (threshold = FDR 5%).

We first evaluated changes in the rectal mucosal proteome associated within the placebo arms in rhesus macaques (Fig. 3). Proteomic data was normalized to total proteomic signal using linear adjustments. Adjusted values were then normalized using log transformation, and normality was confirmed using PP plots. In both placebo gel formulations, significant intra-macaque differences in protein expression were observed between the averaged baseline and the 2 hours post-gel timepoint, with carbopol inducing more changes (76/382 proteins (19.9%), p < 0.017) than HEC (36/382 (9.4%), p < 0.0073) (two-tailed, paired *t* test, Local FDR <5%). Carbopol placebo gel was associated with a larger magnitude of change from baseline, ranging from 4.9 to 4.6 log_2_ fold-change protein abundance, compared to HEC, which ranged from −4.8 to 3.2 log_2_ fold-change protein abundance. No significant changes were observed 24-hour post-placebo gel application, as none passed a 5% FDR threshold.

**Figure 4:**
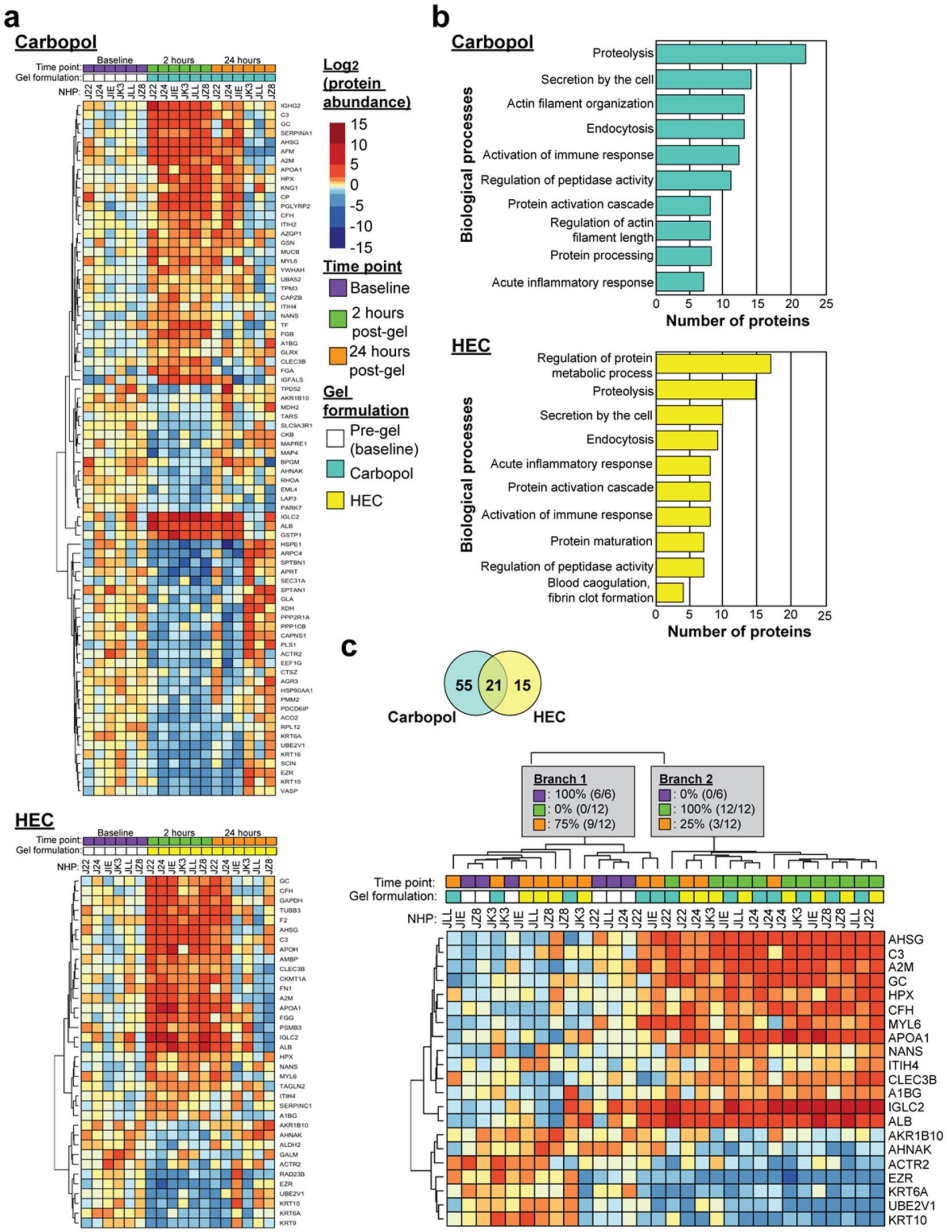
Transient placebo gel-induced effects to the rectal mucosal proteome 2 hours post application in rhesus macaques. Heatmaps display the log_2_-fold change values of protein abundance, with darker colours representing stronger changes. Non-human primate (NHP) identifiers are indicated along the top border of the heatmaps. (**a**) Protein abundance values that were altered 2 hours post-gel application compared to baseline (5% FDR cut off). Baseline, 2 hours, and 24 hours post-gel application time points are indicated in purple, green and orange, respectively. Samples treated with carbopol placebo and HEC placebo are differentiated by turquoise and yellow, respectively. (**b**) Top biological processes associated with altered proteins 2 hours post-placebo gel application, as determined by DAVID v6.8 for carbopol and HEC formulations. Biological functions are listed along the left y-axis and the number of proteins identified in each pathway is indicated along the x-axis. (**c**) Venn-diagram representing the number of altered proteins overlapping between HEC and carbopol placebo gel treatments. Heatmap displaying the 21 proteins commonly affected 2 hours post- HEC and carbopol placebo gel application. The samples clustered into two distinct branches, with all of the samples collected 2 hours post-application clustering together into branch 2.

Hierarchical clustering of differently expressed proteins (FDR < 5%) showed a distinct and transient expression signature associated with samples taken 2 hours post-placebo gel application (Fig. 4a). The DAVID gene ontology tool was used to elucidate known biological functions associated with these proteins (Fig. 4b). Biological functions that were significantly altered (Benjamini-Hochberg corrected Fisher’s Exact Tests) in both HEC and carbopol gel arms included proteolysis (carbopol, p = 1.38E-3, n = 22; HEC, p = 7.12E-4, n = 15), secretion by the cell (carbopol, p = 2.52E-5, n = 14; HEC, p = 2.93E-5, n = 10), endocytosis (carbopol, p = 3.18E-3; n = 13; HEC, p = 3.64E-3, n = 9), activation of immune response (carbopol, p = 2.99E-3, n = 12; HEC, p = 7.37E-3,n = 8), protein activation cascade (carbopol, p = 7.93E-4, n = 8; HEC, p = 6.5E-6, n = 8), protein maturation/processing (carbopol, p = 6.02E-3, n = 8; HEC, p = 1.91E-3, n = 7), the acute inflammatory response (carbopol, p = 2.94E-3, n = 7; HEC, p = 6.59E-6, n = 8) and regulation of peptidase (carbopol, p = 1.48E-3, n = 11; HEC, p = 8.62E-3, n = 7) activity. Despite a large overlap between biological pathways in the two placebo gel formulations, a few pathways were unique. Pathways unique to carbopol included actin filament organization (p = 1.86E-5, n = 13) and regulation of actin filament length (p = 1.34E-3, n = 8). Regulation of protein metabolic process (p = 1.8E-3, n = 17) and blood coagulation (p = 1.92E-3, n = 4) were unique to HEC gel.

A total of 21 proteins were commonly affected by both HEC and carbopol placebo gel use (FDR < 5%, Fig. 4c). Cluster analysis of these proteins showed a distinct pattern, in which samples clustered according to their collection time point. Branch 1 contained 75% of the 24 hours post-gel application samples and 100% of the baseline samples. Conversely, Branch 2 contained 100% of the 2 hours post-gel application samples and 25% of the 24 hours post-gel application samples. Thus, the protein expression profile in the samples collected 24 hours post-gel application resembled that of the baseline samples. This suggests that after the response at 2 hours, protein levels returned to baseline levels after 24 hours. This distinction further supports our finding that the response seen 2 hours post-gel application was short-lived, disappearing 24 hours post-gel application.

### Griffithsin gel is not associated with changes to the rectal proteome

We next compared the effect of GRFT-containing gels on the rectal mucosal proteome. Compared to placebo, 0.1% GRFT gel did not elicit any significant changes in the rectal mucosa at any time point (FDR < 5%, Fig. 3). In case placebo-associated effects overshadowed any GRFT-related proteome alterations, we compared protein expression in participants using the GRFT gel to pre-gel baseline time points; however, the GRFT-containing gels also did not show any significant effects on rectal protein expression relative to baseline (Supplementary Fig. S6). In order to confirm this finding, we evaluated the expression of proteins involved in specific immune processes, including proteolysis, complement, the acute inflammatory response, epithelial cell differentiation, and wound healing (Supplementary Fig. S7). Complement activation proteins in the 0.1% GRFT-HEC gel arm appeared to cluster based by time point, with the 2 hour post gel-application samples and the 24 hour post-gel application samples separating into distinct groups (Supplementary Fig. S7). However, the statistical significance of this finding could not be determined due to the small sample size. In all other comparisons, there was no observable clustering between the 2 hour and 24 hour post-application time points. Notably, one protein, Myeloperoxidase (MPO), trended towards significance 24 hours post-gel application (p value = 1.73E-4, FDR = 5.23% when placebo and GRFT arms of the carbopol formulation were compared (Fig. 3a). Further analyses of the carbopol formulation show that this effect was likely driven by relatively low MPO levels 24 hours post-placebo gel treatment, skewing the comparison against the 24 hour post-0.1% GRFT gel treatment (Supplementary Fig. S8).

### Structure of the rhesus macaque rectal microbiota

**Figure 5:**
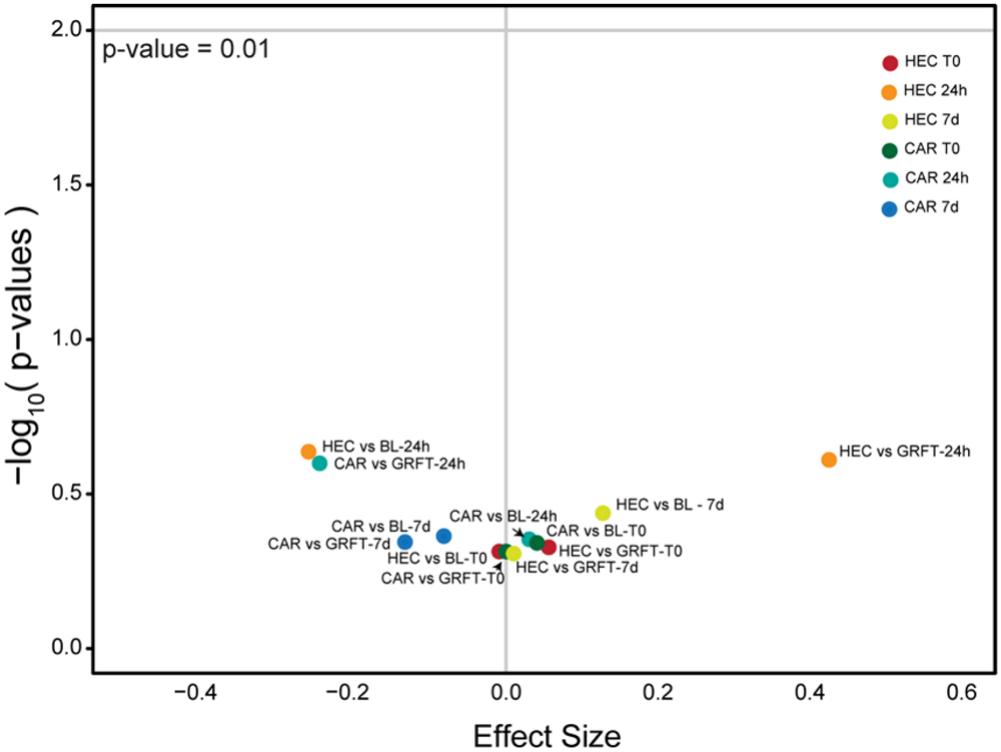
Effects of HEC and carbopol placebo gel or 0.1% GRFT gels on Shannon diversity of rhesus macaque rectal microbiota. Volcano plot displays the effect sizes of compared treatments or placebos on Shannon diversity values on the x-axis and the −log10 p-values on the y-axis. The gray horizontal line indicates p-value of 0.01. There were no significant effects on the Shannon diversity of rhesus macaque rectal microbiota detected.

A total of 81/84 samples were successfully processed (14 samples per animal, Supplementary Tables S1 and S2) and generated a mean number of 58,671 16 S rRNA gene sequences per sample after stringent quality control and assembly. No statistically significant changes in the rhesus macaque rectal microbiota Shannon diversity were observed when comparing placebo (HEC and carbopol) to baseline samples or the 0.1% GRFT gel to placebo samples (Fig. 5).

**Figure 6:**
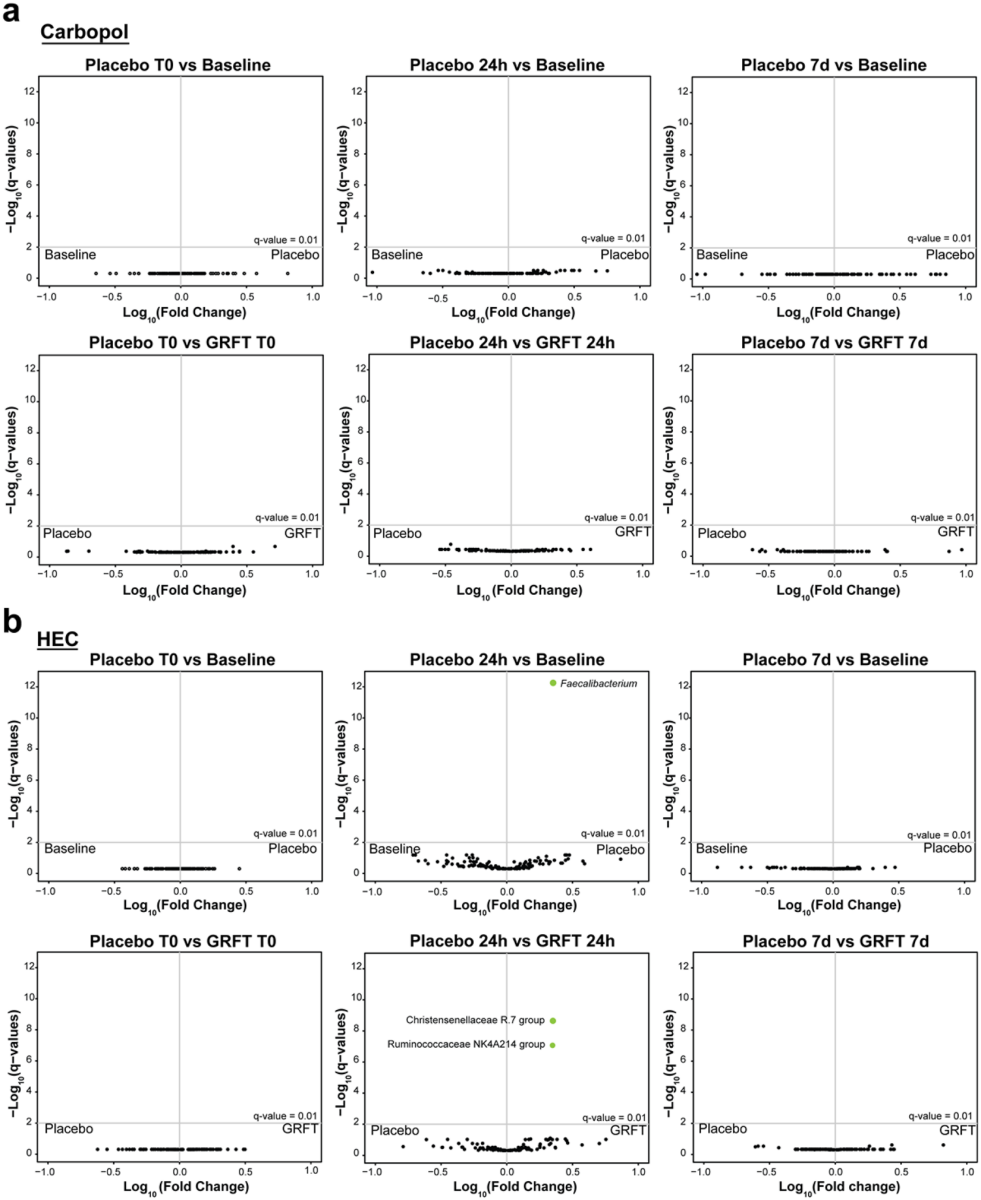
Changes in the rectal microbiota after application of either drug (0.1% GRFT) or placebo using either HEC or carbopol formulations. Volcano plots display the log10-fold change in relative abundances of each detected bacterial taxon along the x-axis and -log10 of the q-values (as determined by Bayesian logistic-normal paired models) along the y-axis. The gray horizontal lines indicate q-value of 0.01. Green points represent taxa for which relative abundances were significantly increased for the treatment (placebo or GRFT) indicated in the right panel of each window. Significant effects were observed 24 h post HEC placebo gel application relative to baseline samples (**b**), and 24 h post-GRFT-HEC gel relative to 24 h post-HEC placebo gel applications (**b**). No significant effects were observed in any other bacterial taxa or treatments.

The effects of placebo (HEC and carbopol) or GRFT-gel application on specific rectum-associated bacterial taxa were evaluated. For each comparison, estimates of the mean of log ratios of taxa relative abundances in each treatment and control were modeled (Fig. 6). A significant increase in the relative abundance of *Faecalibacterium* was observed 24-hours post HEC placebo gel application relative to baseline samples (q-value 5.5 × 10^−13^, Table 1). However, this effect was not observed 7-days post HEC placebo gel application. In addition, a significant increase in the relative abundances of *Christensenellaceae* R.7 and *Ruminococcaceae* NK4A214 were detected 24-hours after application of GRFT-HEC gel relative to 24-hours post HEC placebo gel application (q-values 2.2E-09and 8.4E-08, respectively Table 1). No statistically significant changes in the rectal microbiota were observed in the other comparisons tested (Fig. 6). Notably, carbopol placebo or GRFT-carbopol gel application had no statistically significant effects on the structure and composition of the rectal microbiota.

**Table 1:** Taxa for which relative abundances significantly changed between indicated conditions.

	**Effect Size**	**p-value**	**q-value**	**n**	**Median proportion (Treatment)**	**Median proportion (Control)**	**Median proportion** **(7 d)**
**HEC Placebo 24 h vs Baseline**							
*Faecalibacterium*	0.352	4.21E-15	5.52E−13	6	0.028	0.010	0.018
**GRFT-HEC 24 h vs Placebo 24 h**							
*Christensenellaceae* R.7 group	0.351	1.98E-11	2.24E-09	6	0.020	0.008	0.014
*Ruminococcaceae* NK4A214 group	0.348	1.49E-09	8.42E-08	6	0.008	0.004	0.009

*Faecalibacterium* relative abundances were significantly increased 24 h post HEC placebo gel application (Treatment) relative to baseline samples (Control). Relative abundances of *Christensenellaceae* R.7 and *Ruminococcaceae* NK4A214 were significantly increased 24 h after application of GRFT-HEC gel (Treatment) relative to 24-hours post HEC placebo gel application (Control).

## Discussion

This study was performed to evaluate the effects of 0.1% GRFT gel on the rectal mucosal proteome and microbiome when formulated in either HEC or carbopol. Importantly, this pilot study showed that 0.1% GRFT gels did not associate with any significant alterations to the rectal mucosal proteome when compared to matched placebo gel formulations. Additionally, small, but significant increases in relative abundances of *Ruminococcaceae* NK4A214 and *Christensenellaceae* R-7, both of which are low-abundant members of the rhesus macaque rectum-associated microbiota, were detected. This effect disappeared 7 days post gel application. In both the proteome and the microbiota, significant changes were associated with placebo gel application. This initial pilot study using a model of non-human primates suggests that placebo gels may not be completely inert with respect to effects on rectal mucosa and microbiota.

The antiviral activity of lectins is due to their ability to inhibit viral replication through interactions with the glycans on the viral envelope^41^. Importantly, the oligosaccharides of the viral envelope are synthesized and attached by host enzymes, and thus, similar oligosaccharides may be found on the surface of host cells. Consequently, lectins used as antiviral therapies may have off-target effects on host cells. Specifically, lectins are known to agglutinate cells, and many lectins have demonstrated mitogenic activity^42,43^. For these reasons, there is concern that lectins could have potential off-target side effects in the host. Additionally, as foreign proteins, lectins may induce acute immune responses in the host. These responses are important in the context of HIV infection, in which increased immune activation may lead to increased HIV target cell recruitment at mucosal surfaces. Previous studies of GRFT show that it has a promising safety profile, with no cytotoxicity in several different cell lines, including cervical (End1/E6E7, Ect1/E6E7, CaSki), fibroblast (3T3) and dendritic (moDC) cell lines^32,33^. These studies also showed that GRFT had no mitogenic activity in cervical cell lines, or in human PBMCs^32,35^. Additionally, GRFT induced little to no changes in inflammatory cytokine profiles in cervical cell lines and in human cervical explant tissue^32,35^. *In vivo* studies showed that GRFT was non-irritating and non-inflammatory in the rabbit vaginal irritation model and that GRFT had no effect on the mucosal immune response in mice^33,35^. The results from the current study support the safety of GRFT, as we found no association of GRFT gel formulations with any significant alterations to the rectal mucosal proteome when compared to matched placebo gel formulations.

We observed that application of HEC and carbopol placebo gels associated with transient changes in the acute inflammatory response and activation of the immune system. This may be important in the context of HIV infection, as inflammation increases risk for transmission^20^, although the importance of these molecular pathways at mucosal surfaces to viral susceptibility is unknown. Notably, a study in a macaque model by *Vishwanathan et al*. showed that the application of a rectal lubricant induced a transient cytotoxicity, but did not increase risk of SHIV infection^44^. While many studies have documented the safety of vaginal and rectal placebo gels containing hydroxyethylcellulose (HEC)^45–47^, others have reported undesirable effects of HEC-containing gels on the vaginal and rectal mucosa^26,48–50^. There have also been many studies conducted on gels formulated with Carbopol 974 P showing various results^51–53^. It is important to acknowledge the compositional differences between the HEC- and carbopol- formulated gels used in our study and the gels used in the studies mentioned above. For this reason, it is difficult to make direct comparisons between our study and others.

Microbiota analyses of samples collected 24 hours post-HEC placebo gels showed a ~2 fold increase in the relative abundance of *Faecalibacterium* spp., which are anaerobic, broadly distributed members of the gut microbiome^54^. *Faecalibacterium* are also considered important members of a healthy gut, recognized as butyrate producers, a small chain fatty acid shown to have anti-inflammatory properties. The small but statistically significant increase in relative abundance of this taxon suggests that HEC placebo gel does not have a negative effect on the structure of the gut microbiota, but it may not be inert. In addition, the effect was only observed 24 hours-post HEC application, and disappeared by day 7. No effect on the rhesus macaque rectal microbiota was observed after application of carbopol placebo gels or 0.1% GRFT carbopol gel at any of the time points tested. Notably, recent studies found an enrichment of genera from the *Prevotella* enterotype, in MSM compared to non-MSM. They suggest that this difference may be associated with certain sexual preferences of MSM, including the common use of lubricant gels^55^. We did not observe any significant alterations in the relative abundance of Prevotella associated with gel application in rhesus macaques.

Our study had a few limitations. First, the power of our study was restricted by the small sample size. Second, it is difficult to determine whether the inflammatory processes associated with placebo gel application are due to the chemical components of the placebo gels, or rather, due to the mechanical application procedure itself. With only a single layer of columnar epithelium, the rectal mucosal barrier is relatively fragile, and is possible that the application of any gel may cause injury and induced transient inflammatory events. Additionally, the gel products used in this study contained additional gel excipients, such as glycerine, EDTA and preservatives, which may be associated with a biological response in rectal mucosa. Unfortunately, without proper control groups, we are unable to assess the importance of these effects. Future studies should include a mechanical control (i.e. sham gel application) and positive gel controls, such as imiquimod or N-9. Finally, the samples were collected without aid of an anoscope which may have contributed to more variability in sample collection and limiting sensitivity to detect mucosal changes.

In conclusion, this multi-platform study incorporating proteomics and microbiome techniques provided comprehensive information of the rectal mucosal environment at the systems level, providing an additional toolset for pre-clinical microbicide safety. Furthermore, the transient inflammatory alterations to the proteome observed with placebo gel use demonstrates that these gels may not be completely inert and will serve as important parameters to monitor in future safety studies. Overall, compared to placebo, 0.1% Griffithsin microbicide gel did not elicit any significant effects to the mucosal proteome and minor effects to the microbiome, suggesting tolerability and suitability as a microbicide candidate.

## Methods

### Gel products

Carbopol-formulated 0.1% GRFT and placebo gels contained Carbopol 974 P, methylparaben, propylparaben, methionine, Ethylenediaminetetraacetic acid (EDTA) and purified water. HEC-formulated 0.1% GRFT and placebo gels contained hydroxyethylcellulose, methylparaben, propylparaben, methionine, and purified water.

### Rectal swab collection

Study design details are provided in the results section and an overview of the pre-clinical trial, which involved six Rhesus Macaques (*Macaca mulatta*, n = 6), is provided in Fig. 1. Each gel application was preceded by collection of pre-gel (T0) rectal swabs and followed by collection of post-gel rectal swabs (2 hours and 24 hours post-gel application for proteomic analysis; 24 hours and 7 days post-gel application for microbiota analysis). Swabs for proteomic analysis (Weck-cel swabs or Merocel sponges, Beaver Visitec Intl Ltd, Fisher Scientific #NC0240644) were pre-wet with cold PBS and inserted into the rectum. Swabs for proteomics were then removed after 5 minutes and excess feces were scraped off. These swabs were then placed in a Costar Spin-X centrifuge tube filter (Sigma Cat. #CLS8160) and eluted with PBS by centrifugation at 4 °C for 30 minutes (16000 rpm). Rectal swab eluates (RSE) were placed into new Spin-X tubes and stored at −80 °C until further proteomic processing. Swabs for microbiota analysis were collected using Copan ESwab system (Copan #480 C) by inserting and rotating the swab three times in the rectum. The swab was then stored in 1 mL of Amies transport medium and stored at −80 °C until processing.

### Mass spectrometry analysis of rectal swab eluates using label-free proteomics

Rectal swab samples (n = 96) from six macaques across the 16 time points were collected, processed and digested into peptides. Remaining cellular debris was removed by centrifugation at 23,000 g for 30 minutes. Equal volumes of RSE samples were denatured by adding 600 μl 8 M Urea Exchange Buffer and then digested using filter-aided sample preparation, described previously^56,57^. Salts and detergents were removed using reverse-phase liquid chromatography (high pH RP, Agilent 1200 series micro-flow pump, Water XBridge column) using a step function gradient eluted into a single sample. Peptide concentration was determined using a FluorProphile® Protein Quantification kit (Sigma-Aldrich, St. Louis, MO) and RSE samples (1 μg peptide per sample) were analysed using an Easy nLC nanoflow LC system (Thermo Fisher Scientific, Waltham, MA) connected in line with an Linear Trap Quadrupole (LTQ) Orbitrap Velos mass spectrometer (Thermo Fisher Scientific, Waltham, MA) as described previously^57^. Briefly, tryptic peptides were loaded onto a low-pH reversed-phase column (0.075 × 15 cm), eluted over a 120-min gradient (2%–30% acetonitrile, 0.1% formic acid), and nanosprayed into the LTQ Orbitrap Velos. The survey scans (MS1) were acquired in the Orbitrap at resolution of 60,000, and data-dependent acquisition was used to select the top 10 abundant peptide ions for fragmentation by collision induced dissociation. The peptide fragment ion scans (MS2) were acquired in the LTQ.

### Analysis of mass spectrometry data

Progenesis Software (v4.0; Nonlinear Dynamics, Durham, NC) was used to process raw MS spectra. One reference sample, which included equal volumes from each sample, was used to align sample spectra automatically, with manual revision for errors. The reference was run every 10 samples on the mass spectrometer, and these technical replicates were used to determine the reproducibility of the MS data. Peptides with a charge state between +2 and +7 and a retention time between 10 and 125 min were retained. Filtered spectra were annotated using Mascot software (v2.4; Matrix Science, Boston, MA) using the UniProtKB/SwissProt (2012, v3.87) database for human and bacterial proteins. Searches were performed with the following criteria: carbamidomethyl (C) fixed modifications, oxidative (M) variable modifications, a fragment ion mass tolerance of 0.5 Da, a parent ion tolerance of 10 ppm, tryptic enzyme digestion with a tolerance of one missed cleavage, and a decoy database. Search results were imported into Scaffold (v4.4.1.1; Proteome Software, Portland, OR) to filter protein identifications (80% confidence for peptide identification, 95% confidence for protein identification, and a minimum of two unique peptides identified per protein). Only proteins matched to human were included in downstream analysis. Feature detection and quantification were all performed using default settings from the software.

### DNA extraction and 16 S rRNA gene amplication from rectal swabs

The swabs were thawed on ice, and 300 µl of Amies transport medium containing vaginal secretion were processed using the MoBio Microbiome kit automated on a Hamilton Microlab STAR robotic platform after a bead-beating step on a Qiagen TissueLyser II (20 Hz for 20 min) in 96 deep well plate. Amplification of the V3-V4 regions of the 16 S rRNA gene was performed using a two step-PCR in which the sample specific barcode is added during the second PCR, to maximize target amplification. The first PCR used the short 16 S rRNA gene specific primers 319 F (ACACTGACGACATGGTTCTACA[0–7]**ACTCCTRCGGGAGGCAGCAG**) and 806 R (TACGGTAGCAGAGACTTGGTCT[0–7]**GGACTACHVGGGTWTCTAAT**) where the underlined sequence is the Illumina sequencing primer sequence and [0–7] indicate the presence of an heterogeneous pad sequence to improve sequencing quality^58^, for a total of 20 cycles. This first step was followed by 10 cycles with primers H1 (AATGATACGGCGACCACCGAGATCTACACNNNNNNNNACACTGACGACATGGTTCTACA) and H2 (CAAGCAGAAGACGGCATACGAGATNNNNNNNNTACGGTAGCAGAGACTTGGTCT) where NNNNNN indicates a sample specific barcode sequence and the underlined sequence corresponds to the Illumina sequencing primer for priming to the first step amplicon. This second step extends the amplicon with the Illumina required adaptor sequences and the sample specific dual barcode system^58^. Amplicons were visualized on a 2% agarose gel, quantified, pooled in equimolar concentration and purified prior to loading on an Illumina HiSeq 2500 (San Diego, CA, USA) modified to generate 300 bp paired-end reads. Four extraction and four PCR negative controls were processed in parallel. Additionally, 14 positive controls composed of a mixture of fecal and vaginal biological specimen were processed and sequenced in parallel to the study samples as per the laboratory standard protocol (Supplementary Table S3).

### Bioinformatics methods for microbiota analysis

#### Quality controls

The sequences were demultiplexed using the dual-barcode strategy, the mapping file generated on the robotic platform and split_libraries_fastq.py, a QIIME-dependent script^59^. The resulting forward and reverse fastq files were split by sample using seqtk (https://github.com/lh3/seqtk), and primer sequences were removed using TagCleaner (0.16) (Schmieder R *et al*. 2017 BMC Bioinformatics). Further processing followed the DADA2 Workflow for Big Data and dada2 (v. 1.5.2) (https://benjjneb.github.io/dada2/bigdata.html,60). Forward and reverse reads were each trimmed using lengths of 255 and 225 bp, respectively, and filtered to contain no ambiguous bases, minimum quality score of 2, and required to contain less than two expected errors based on their quality score. The relationship between quality scores and error rates were estimated for both sequencing runs to reduce batch effects arising from run-to-run variability. Reads were assembled and chimeras for the combined runs removed as per dada2 protocol.

Taxonomy was assigned to each amplicon sequence variant (ASV) generated by dada2 using the SILVA v128 database and the RDP naïve Bayesian classifier as implemented in the dada2 R package^60,61^. Read counts for ASVs assigned the same taxonomy were summed for each sample. A table including total sequence count for each taxon and for each sample was generated and used for statistical analyses (Supplementary Tables S2 and S3).

### Statistical analysis

#### Proteomics

Progenesis Software was used to normalize protein abundances in each sample to total ion current. Technical variability was assessed using technical replicates of a reference sample (consisting of equal amounts of peptide from each sample) run once every 10 samples on the mass spectrometer. Proteins with a coefficient of variance below 25% between these mixes were retained and those greater than 25% were removed from the downstream analysis. All samples were further normalized to the median protein abundance using linear regression. Outlier samples were identified as having a normalized protein abundance that was greater than 1.5 times the interquartile range of the median normalized abundance of proteins identified across all samples. A total of 3/96 samples were identified as outliers, but were kept in downstream analysis due to the longitudinal nature of the study. Samples and protein identifications were subject to differential expression analysis. For each macaque, protein levels from all baseline time points (n = 4) were averaged. Protein levels from samples collected at T0, 2 h post gel and 24 h post-gel application were then log2 transformed, normalized to their respective baseline average, and analysed using two-tailed, paired *t* tests to determine changes in protein expression throughout the trial. Q-q plots were generated and assessed to ensure the assumptions of normality applied to our data set and that the use of parametric statistics on our small sample size was acceptable. Notably, protein abundances were not normalized to their respective baseline average in cluster analyses that included baseline values. This was done in order to avoid skewing the clustering algorithm where Pearson’s correlation distance metric and complete linkage were applied. G*Power 3.1 was used to calculate the power of this study. Our study was powered to confidently detect protein changes greater than |2.3 Log2 FC| to retain an experimental power of 80%. Proteins determined to be significantly differentially expressed were those that passed a local false discovery rate (FDR) of 5% using the Benjimani-Hochberg method to adjust for a total of 382 protein comparisons. Functional annotation analysis and pathway analysis of these proteins were performed using DAVID (Database for Annotation, Visualization, and Integrated Discovery, v6.8) and Ingenuity Pathway Analysis (IPA, QIAGEN Inc., https://www.qiagenbioinformatics.com/products/ingenuity-pathway-analysis); statistical enrichment values were calculated according to default software parameters^62,63^. Right-tailed Fisher’s Exact Tests (Benjamini-Hochberg corrected) were used to calculate the probability that the association between each protein in our experimental dataset compared to the manually curated and annotated datasets were random.

#### Microbiota

Taxa were filtered before analysis if observed at frequencies of 10^−5^ study-wide or if observed in fewer than 25% of samples study-wide or, within each comparison, present in at least 4 subjects. To test for the effect of treatment on community diversity, the mean value of Shannon diversity was compared using a Bayesian paired normal-Laplace model fitted to the logit transformed Shannon diversity data. The logit transformation was applied so that the distribution of transformed values was normally distributed. The paired normal-Laplace model had the following structure:

$${\rm{yT}}\_{\rm{i}} \sim \mathrm{norm}(\mathrm{muT}\_{\rm{i}},\,\mathrm{sigmaT})\mathrm{yC}\_{\rm{i}} \sim \mathrm{norm}(\mathrm{muC}\_{\rm{i}},\,\mathrm{sigmaC})$$

where yT_i and yC_i are the transformed Shannon diversity values of the i-th treatment and control samples, respectively. Under this model it is assumed that the difference in the mean Shannon diversity values of treatments and controls (Delta.mu_i = muT_i − muC_i) were sampled from a Laplace distribution (double exponential); Delta.mu_i ~ Laplace(mu, sigma). For each condition, 3 chains with 10,000 iterations and thinning of 10 were run. Convergence was tested using potential scale reduction statistics.

To test the effect of treatment on the relative abundance of each detected taxon, a Bayesian logistic-normal paired model was built as follows:

$${\rm{yT}}\_{\rm{i}} \sim \mathrm{bin}(\mathrm{nReadsT}\_{\rm{i}},\,{\rm{pT}}\_i)$$

$${\rm{yC}}\_{\rm{i}} \sim {\rm{bin}}({\rm{nReadsC}}\_{\rm{i}},\,{\rm{pC}}\_{\rm{i}})$$

where yT_i and nReadsT_i are the i-th treatment sample’s sequence count for a given taxon and the sample’s total sequence count, respectively. yC_i and nReadsC_i are the i-th control sample’s sequence count for a given taxon and the sample’s total sequence count, respectively. pT_i and pC_i are the relative abundances of the taxon in the corresponding samples. To account for measurement uncertainty of low abundant taxa, the positive control dataset was used to build a model of variance of log10 relative abundances of each taxon as a function of its median log10 relative abundances to model pT and pC variables as samples from some true (but unknown) relative abundance tpT and tpC, respectively, and as follows:

$$\mathrm{log}\,10({\rm{pT}}\_{\rm{i}}) \sim {\rm{norm}}(\mathrm{log}\,10({\rm{tpT}}\_{\rm{i}}),\,{\rm{sigmaT}}\_{\rm{i}})$$

$$\mathrm{log}\,10({\rm{pC}}\_{\rm{i}}) \sim {\rm{norm}}(\mathrm{log}\,10({\rm{tpC}}\_{\rm{i}}),\,{\rm{sigmaC}}\_{\rm{i}})$$

where sigmaT_i and sigmaC_i are the standard deviations of the log10 relative abundances corresponding to log10(pT_i) and log10(pC_i) values, respectively. This model estimates the mean of log ratios log10(tpT_i/tpC_i)^64^. For each taxon and each condition, 3 chains with 10,000 iterations and thinning of 10 were run. Convergence was tested using potential scale reduction statistics.

All models were implemented using rstan (v 2.16.2) R package^65^.To account for multiple testing, p-values were adjusted using false discovery rates and statistical significance level was set at q-value = 0.01 (1% FDR).

### Data Availability

The data produced and analysed during this study are available from the corresponding author upon request.

## Electronic supplementary material

Supplemental Figures

Dataset Table S1

Dataset Table S2

Dataset Table S3
