# Supplementary material for: Impact of the griffithsin anti-HIV microbicide and placebo gels on the rectal mucosal proteome and microbiome in non-human primates
Source: Sci Rep. 2018 May 23;8:8059. doi: 10.1038/s41598-018-26313-8 (PMC5966460; doi:10.1038/s41598-018-26313-8)
Supplement: Supplementary file 1 — Supplemental Figures [file 41598_2018_26313_MOESM1_ESM.docx]

**Impact of the griffithsin anti-HIV microbicide and placebo gels on the rectal mucosal proteome and microbiome in non-human primates**

Lauren Girard^1, 2^, Kenzie Birse^1, 2^, Johanna B. Holm^3,^4, Pawel Gajer^3,4^, Mike S. Humphrys^3,4^, David Garber^5^, Patricia Guenthner^5^, Laura Noël-Romas^1, 2^, Max Abou^1^, Stuart McCorrister^6^, Garrett Westmacott^6^, Lin Wang^7, 8^, Lisa C. Rohan^7, 8^, Nobuyuki Matoba^9,10,11^, Janet McNicholl^5^, Kenneth E. Palmer^9, 10,11^, Jacques Ravel^3,4^, Adam Burgener^1, 2,12 *^


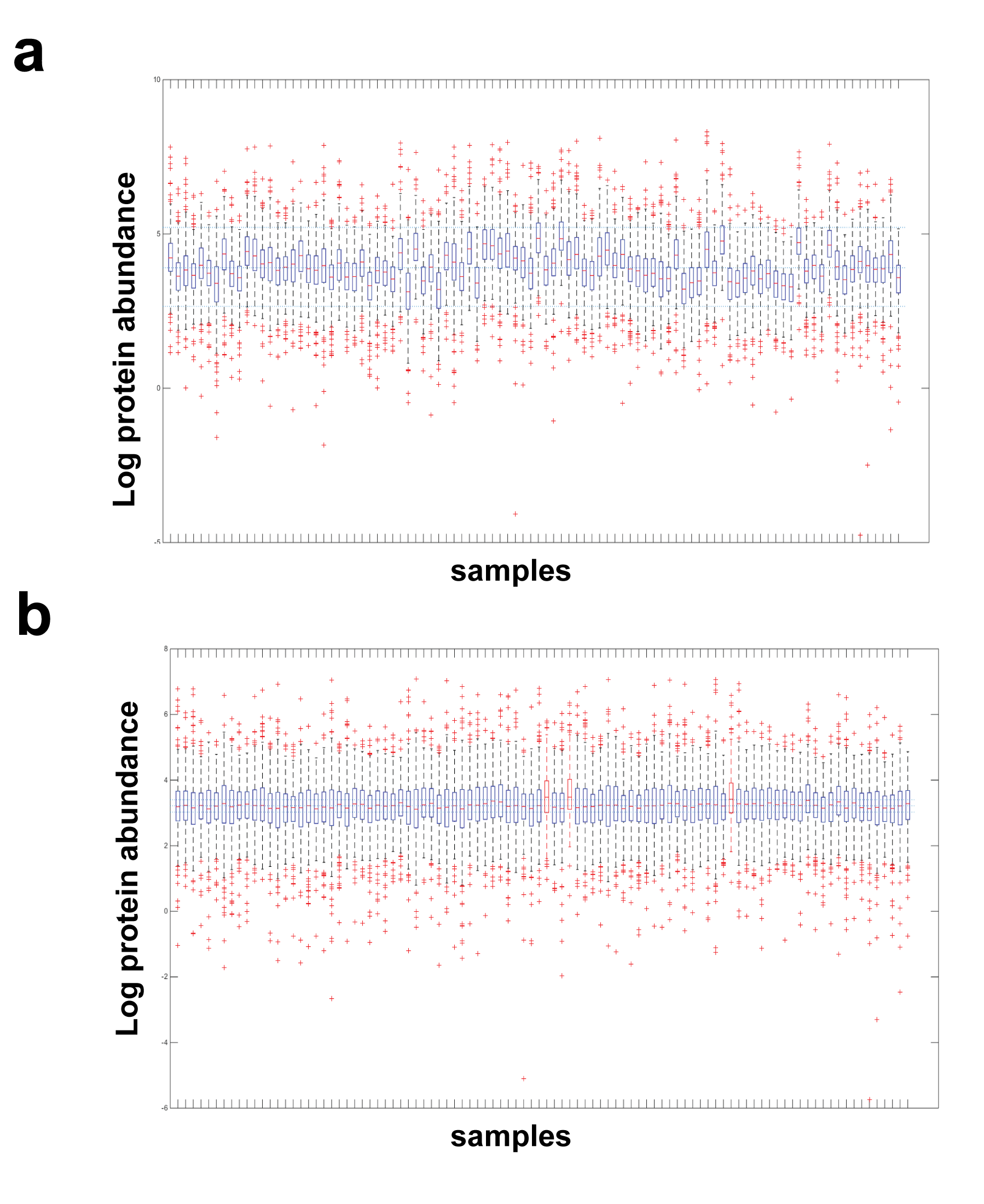


**Supplementary Figure S1:** Protein abundance distribution plots of rectal mucosal samples. Box and whisker plots display the median, interquartile range and minimum and maximum of the Log_10_ normalized abundance values for identified proteins in each sample. Due to the variability between samples (**a**, pre-adjustment data), a second normalization step was performed. The median normalized abundance of total protein across all samples was calculated. Outlier samples were determined as having median total protein abundance greater than 1.5 times the interquartile range of the median normalized abundance of proteins identified across all samples. Three samples were identified as outliers and are indicated in red (**b**).

**
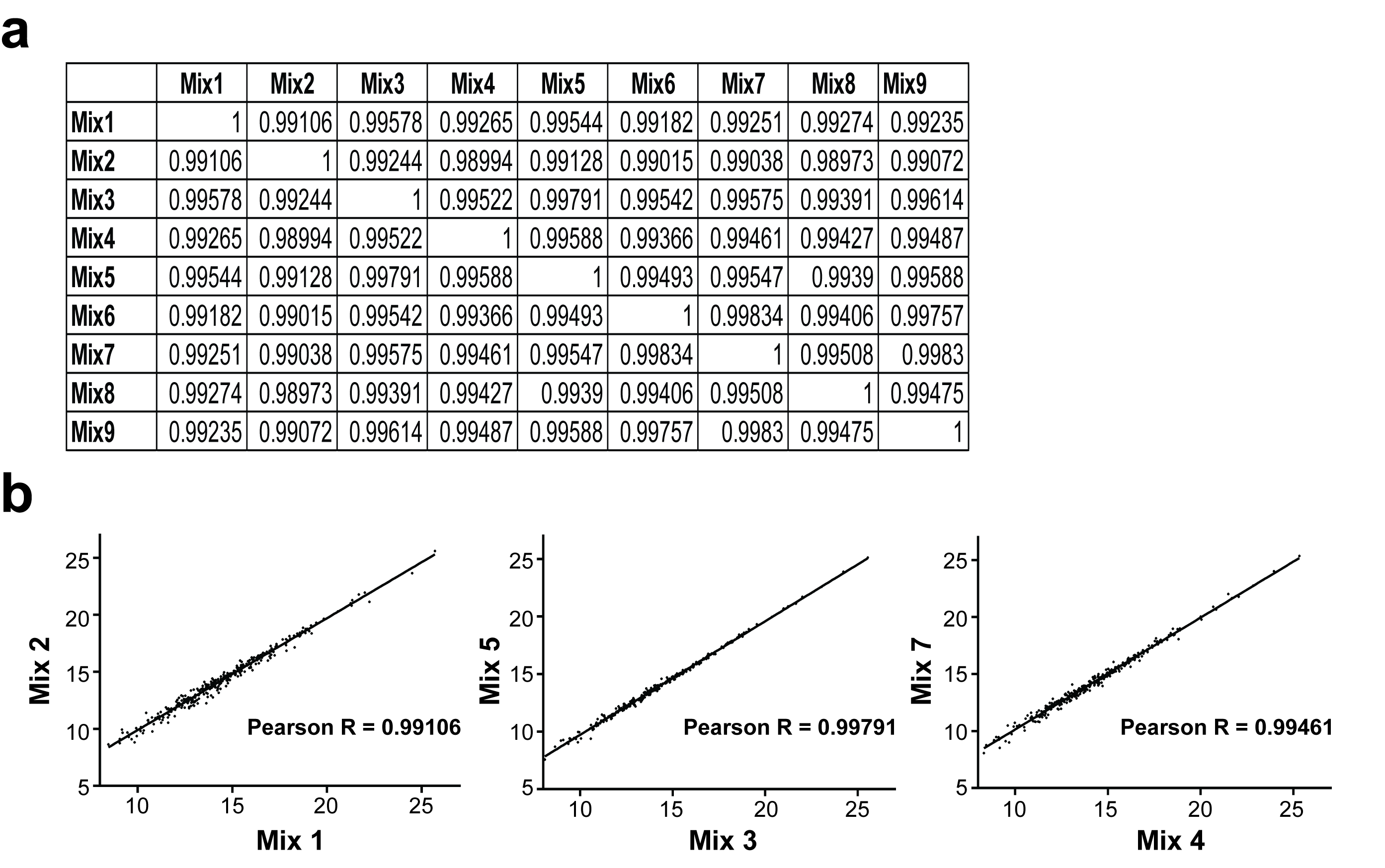
**

**Supplementary Figure S2**: Representative correlation plots showing high reproducibility between mass spectrometry runs of rectal mucosal samples. a) Pearson correlation analysis between technical replicates of a master mix gave r values ranging from 0.989728 to 1; b) Representation of the Pearson correlation analysis between three pairs of mix controls.


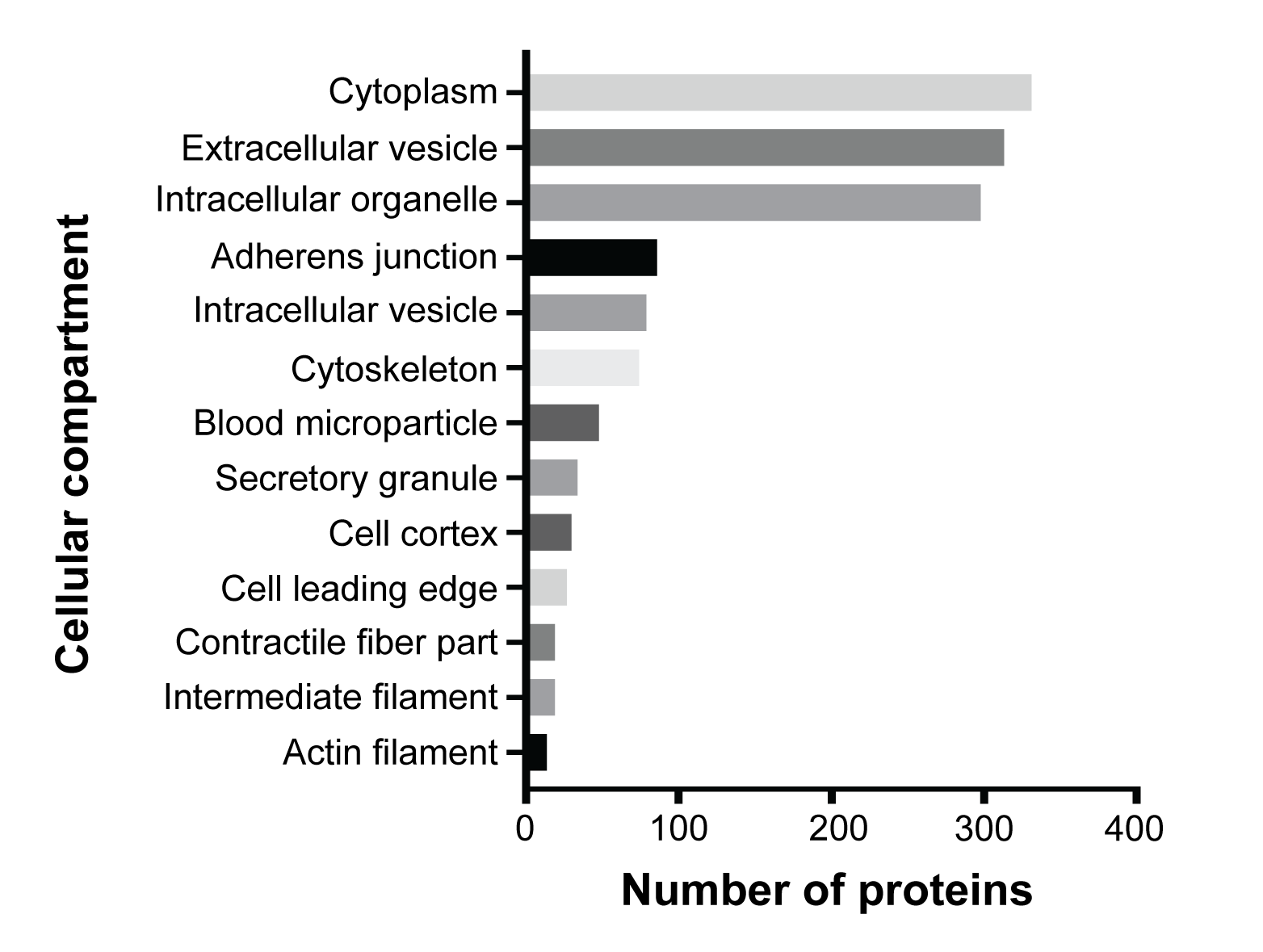


**Supplementary Figure S3**: Bar chart representing the top cellular components in the rectal mucosa of rhesus macaques in our study according to DAVID Functional Annotation v6.8. Cellular components are listed along the left y-axis. The number of proteins in each component is indicated along the x-axis.

**
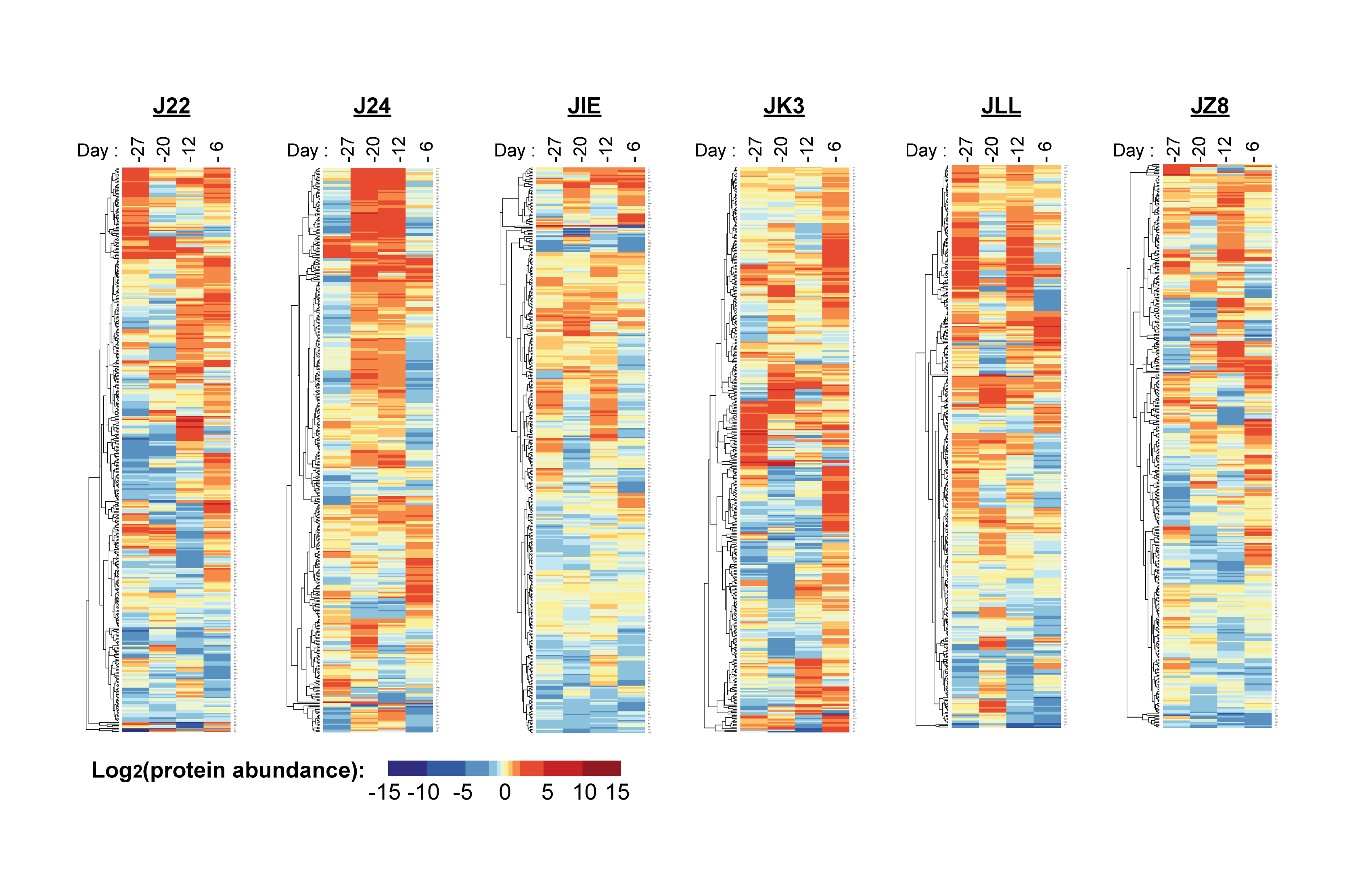
**

**Supplementary Figure S4:** Variation of rectal proteome (382 proteins) in baseline samples collected weekly in individual rhesus macaques. These heatmaps display the log2-fold change values of protein expression in each non-human primate (J22, J24, JIE, JK3, JLL, JZ8) throughout the baseline sampling, with darker colors represent stronger changes.


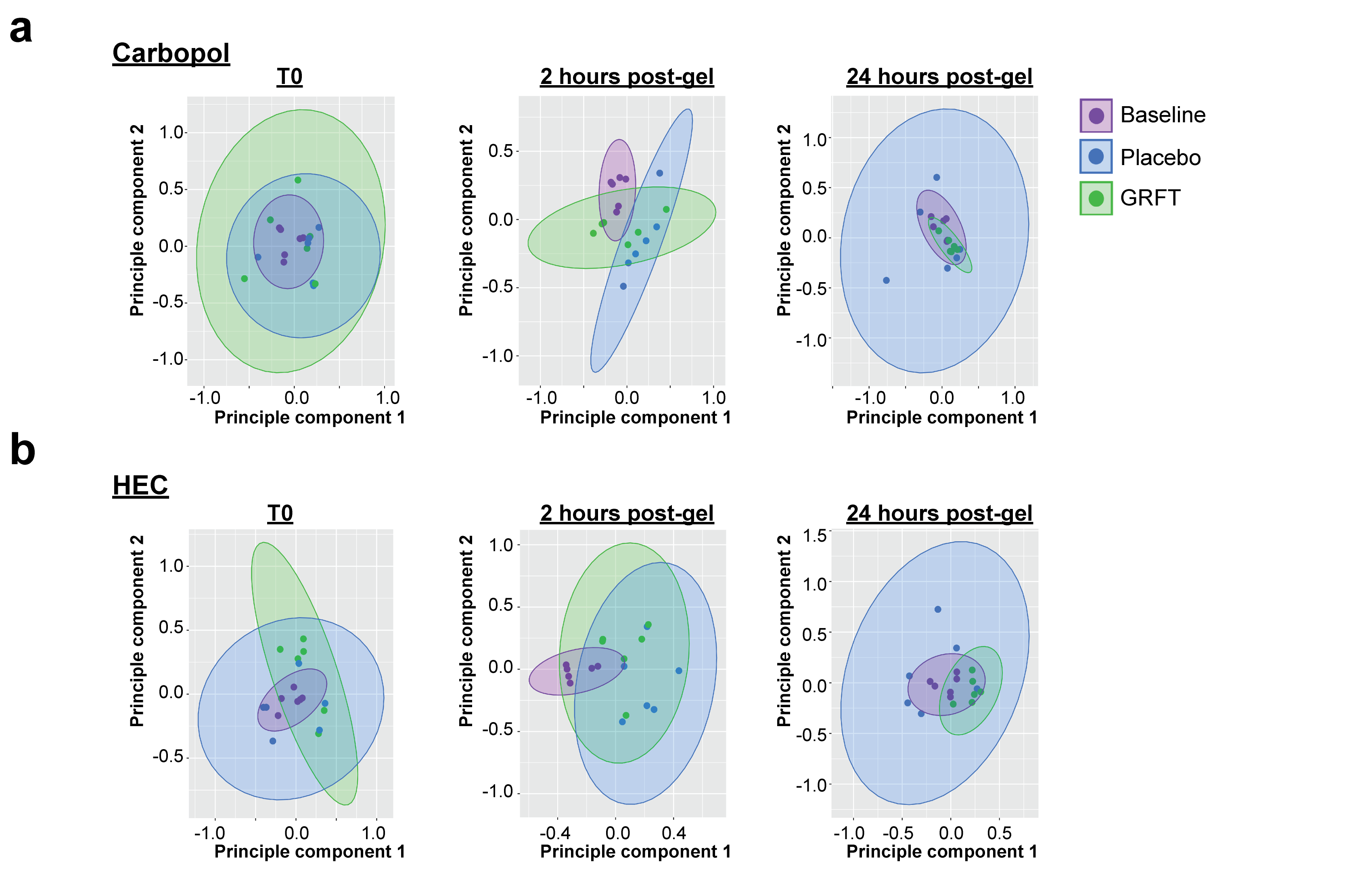


**Supplementary Figure S5**: Principle component analysis of all proteins prior to gel application (T0) 2 hours post-gel application and 24 hours post-gel application for the carbopol (**a**) and hydroxyethylcellulose (HEC) (**b**). Baseline, placebo-treated and GRFT-treated samples are indicated by purple, blue and green, respectively.

**
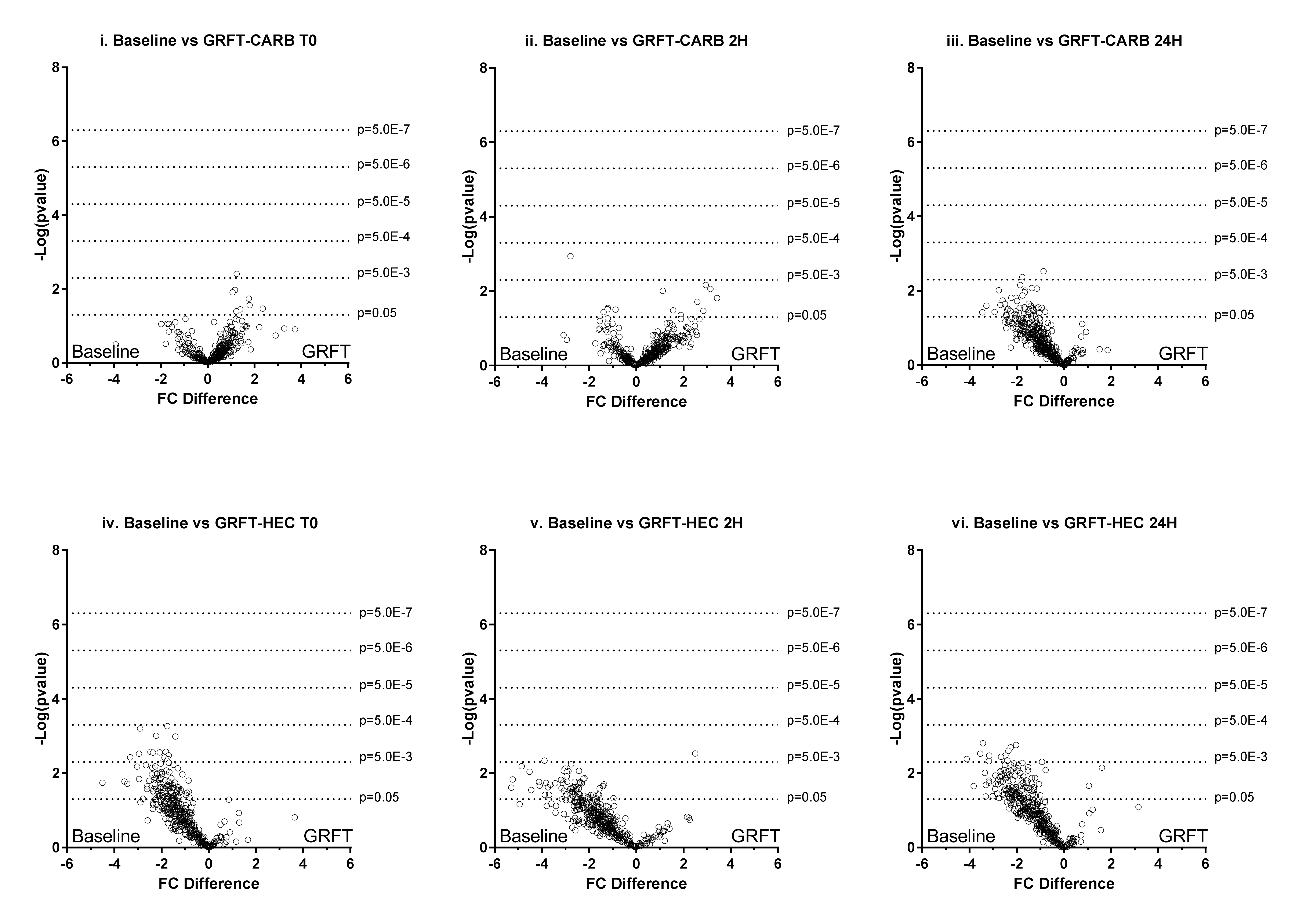
Supplemental Figure S6: Changes to the rectal mucosal proteome after application of 0.1% GRFT formulations relative to pre-gel, baseline time points**. Volcano plots display the log2-fold change values of protein expression along the x-axis (GRFT-Baseline) and -log p-value (as determined by paired *t* tests) along the y-axis. Carpbopol-based GRFT (GRFT-CARB) gel use showed no significant alterations to rectal protein expression immediately (i), 2 hours (ii), or 24 hours (iii) following GRFT exposure. Protein expression after HEC-based GRFT gel (GRFT-HEC) exposure showed a trend toward decreased protein expression relative to baseline; however no proteins changes passed multiple hypothesis testing correction at any post-gel time points (iv, v, vi).


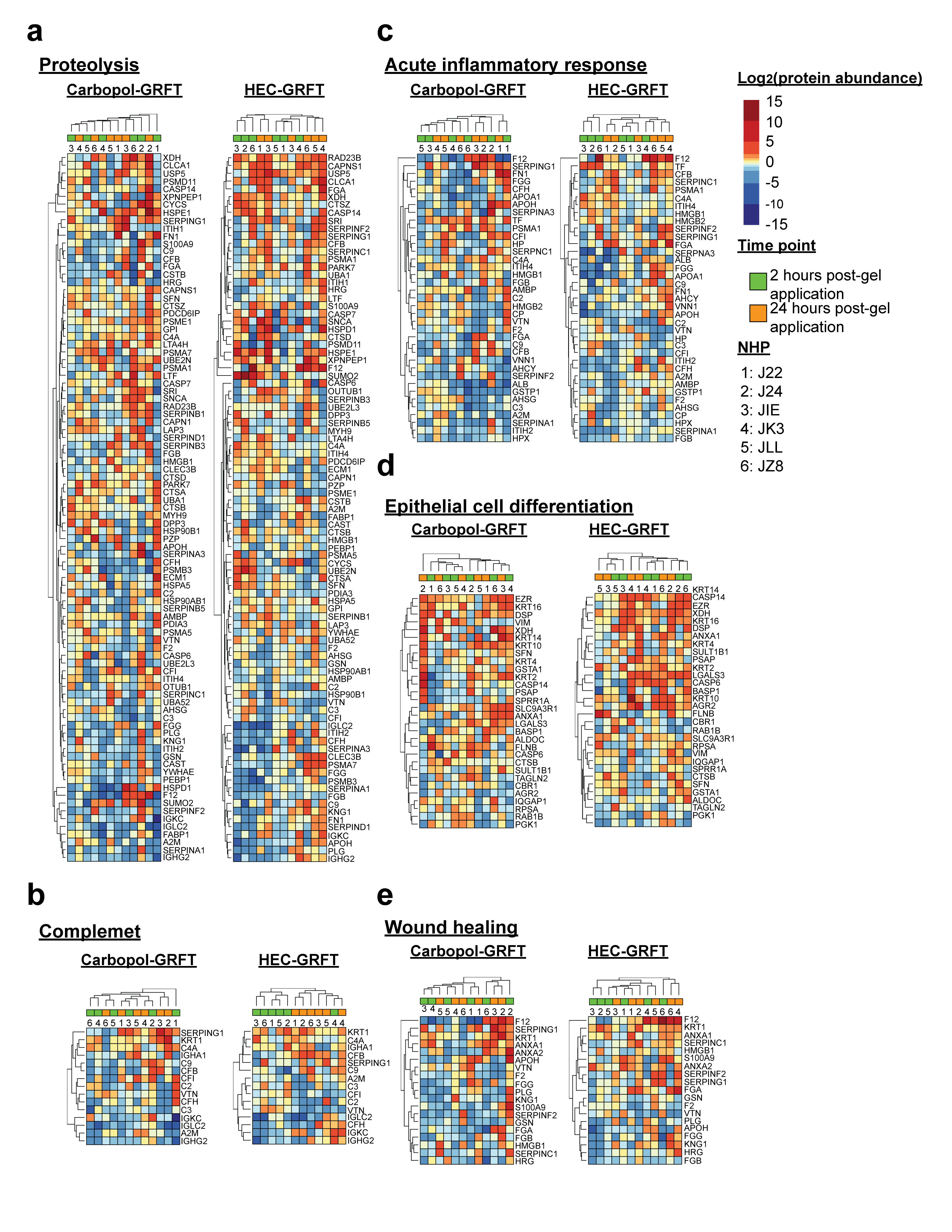


**Supplementary Figure S7:** Hierarchical clustering of proteins involved in various immune processes associated with intra-rectal application of 0.1% GRFT gels. Clustering parameters were set as Pearson’s correlation distance metric and complete linkage. These heatmaps display the log_2_-fold change values of protein abundance, with darker colors representing stronger changes. Individual NHPs are differentiated by numbers along the top border of each heatmap (1:J22; 2:J24; 3:JIE; 4:JK3; 5:JLL; 6:JZ8). Gene names are indicated along the right border. a) Proteolysis; b) Complement; c) Acute inflammatory response; d) Epithelial cell differentiation; e) Wound healing. Carbopol and HEC 0.1% GRFT gel application are indicated in turquoise and yellow, respectively. Samples collected 2 hours and 24 hours post-gel application are indicated in green and orange, respectively.


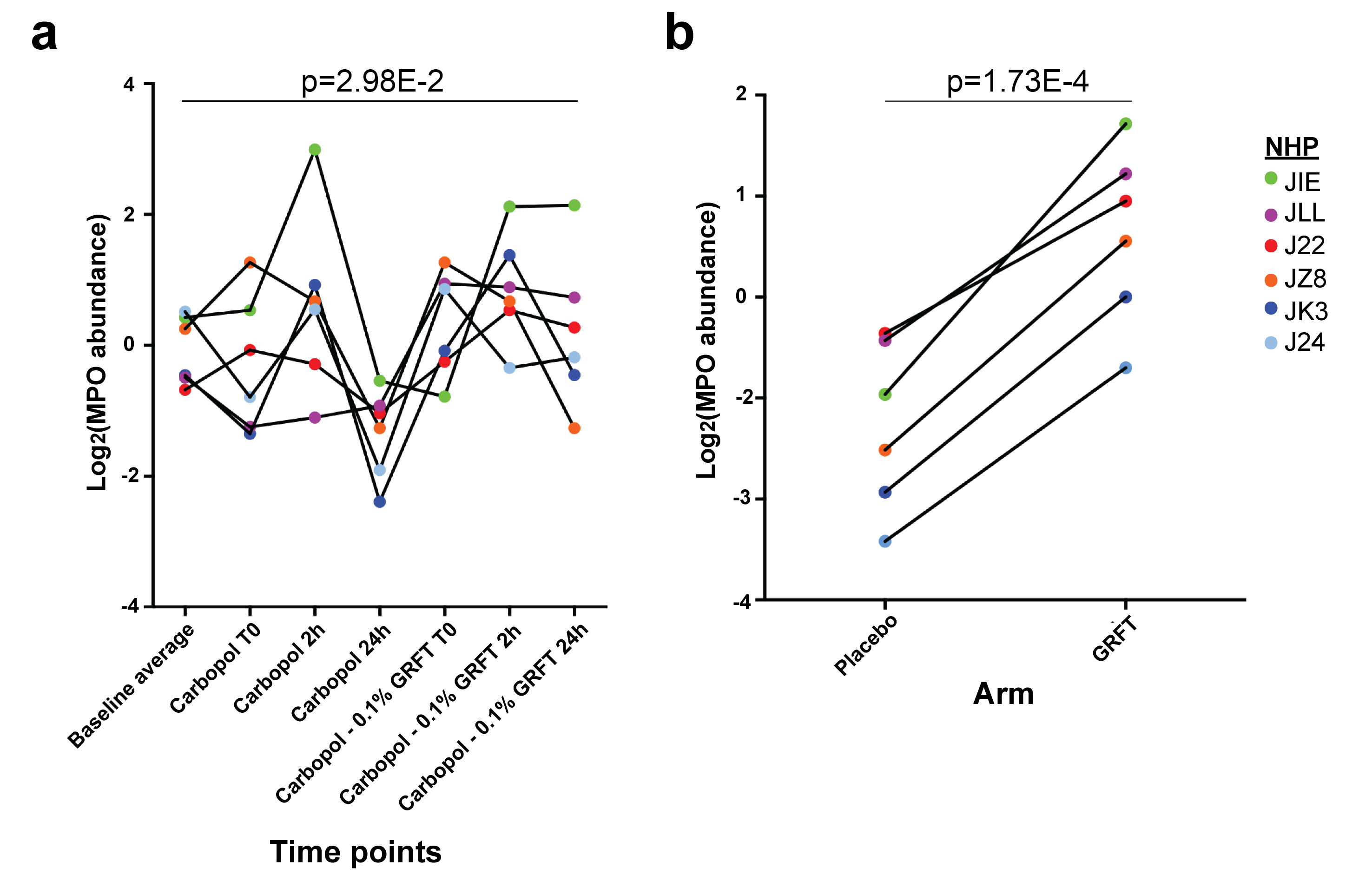


**Supplementary Figure S8:** Changes in Myeloperoxidase (MPO) abundance pre- and post-gel application. (a) MPO abundance varied over time in the carbopol formulation, with a sharp drop in abundance 24 hours post-placebo gel application (p=2.98E-2, Repeated measures ANOVA). (**b**) A significant difference (P=1.73E-4; two-tailed, paired *t* test) is seen when comparing MPO levels from 24 hours-post placebo treatment compared to 24 hours post 0.1% GRFT treatment However, this difference appears to be driven by low MPO levels post-placebo gel treatment (**a**) instead of an increase in MPO due to 0.1% GRFT gel treatment. This comparison did not pass multiple comparison correction which was set to a false discovery rate below 5%.
